# Evaluation of Diagnostic Utility of the Immunohistochemical Markers in the Accurate Diagnosis of Thyroid Neoplasms: A Retrospective Study in a Tertiary Care Hospital of Pakistan

**DOI:** 10.7759/cureus.20953

**Published:** 2022-01-05

**Authors:** Kaleemullah Badini, Saira Fatima, Sajjad Ali Khan, Zafar Suchal, Najmul Islam

**Affiliations:** 1 Department of Medicine, Section of Endocrinology, Aga Khan University Hospital, Karachi, PAK; 2 Department of Pathology and Laboratory Medicine, Section of Histopathology, Aga Khan University Hospital, Karachi, PAK; 3 Department of Medicine, Medical College, Aga Khan University Medical College, Karachi, PAK

**Keywords:** developing country, diagnostic utility, immunohistochemical markers, undifferentiated neoplasms, thyroid cancer

## Abstract

Background

Thyroid cancer is the most common endocrine malignancy across the globe and is among the fastest-growing cancers worldwide. Thyroid tumors are divided into differentiated and non-differentiated, with each having further subtypes, with papillary carcinoma being the most common one. Immunohistochemical (IHC) markers’ studies play a crucial role in the accurate diagnosis of thyroid neoplasms. To the best of our knowledge, this topic has been the least researched in Pakistan.

Objectives

This study was designed to determine the diagnostic utility of immunohistochemical markers in the diagnosis of thyroid cancers in correlation with histopathology as the gold standard.

Methods

This retrospective, single-center study was carried out on 124 patients with thyroid cancer treated at our institution. The type of cancer, patient gender, and immunohistochemical markers used in each patient were recorded, and the sensitivity and specificity of the markers used in each tumor case were calculated.

Results

The mean age of patients was found to be 48.5 ± 15.6 years; 56 (45.2%) of the patients were male and 68 (54.8%) were female. Out of the 124 patients, 75 (60.5%) had papillary, 19 (15.3%) had medullary, 16 (12.9%) had anaplastic, and eight (6.5%) had follicular carcinoma, while six (4.8%) had primary thyroid lymphoma. Thyroglobulin was found to be a reliable tumor marker in both papillary and follicular tumors. The cluster of differentiation56 (CD56) negativity was a useful double panel study along with thyroglobulin in the confirmation of papillary carcinomas. Tumor markers used in medullary carcinoma include calcitonin, chromogranin, and synaptophysin. Cytokeratin AE 1 and vimentin were found to be useful for anaplastic tumors, while Ki 67 was a reliable marker for primary thyroid lymphoma.

## Introduction

Thyroid cancer is the most prevalent endocrine cancer, and it is one of the fastest-growing malignancies in the world. Females are three times more likely than males to develop thyroid cancer, and it is the fifth most common tumor among women [1]. It is the ninth most prevalent malignancy, according to the National Cancer Institute's Surveillance, Epidemiology, and End-Result (SEER) database [2].

Proficient knowledge and understanding of thyroid morphology and disease processes have been evolved through several studies [1-3]. It has a wide spectrum of morphology and behavior, with its pathogenesis ranging from common and indolent tumors to aggressive and lethal malignancies [3]. There are four main histopathologic subtypes of thyroid cancer, namely papillary thyroid carcinoma (PTC), follicular thyroid carcinoma (FTC), medullary thyroid carcinoma (MTC), and anaplastic thyroid carcinoma (ATC). Among these variants, PTC is the most common, occurring in 80% of all reported thyroid cancer cases and being the one with the best prognosis [4].

The thyroid gland is composed of two types of cells; follicular cells that generate thyroid hormone and parafollicular cells (C cells) that secrete calcitonin [5]. Differentiated thyroid cancers include PTC and FTC, which are derived from follicular cells. ATC contains undifferentiated cells that develop from follicular cells, whereas MTC is derived from C cells [6].

Major attempts have been made in recent years to find objective techniques to distinguish benign from malignant lesions, mostly employing immunohistochemical (IHC) and molecular markers. With the introduction of molecular gene profiling of malignancies, the diagnostic accuracy of thyroid neoplasms and appropriate patient management have been made possible. Subsequently, more knowledge about immunohistochemistry has aided in the diagnosis and management.

In the classification of thyroid follicular lesions, the histomorphologic diagnosis of thyroid tumors remains the mainstay. On the other hand, immunohistochemical biomarkers may play a supplementary role in the proper classification of tumors that are poorly differentiated and have equivocal histomorphologic features. There is no single marker sensitive enough to provide a definitive diagnosis. Therefore, different panels of combined immunohistochemical markers have been proposed and used [7].

The purpose of our study is to objectively assess the diagnostic utility of immunohistochemical markers in the diagnosis of difficult thyroid lesions such as poorly differentiated and undifferentiated neoplasms. Furthermore, we aim to correlate the association of immunohistochemical markers with histopathology, which is the gold standard in the diagnosis of thyroid cancers. The subject has been studied internationally, but there is a paucity of data in this part of the world.

## Materials and methods

Study design and participants

This was a retrospective single-center observational study carried out in a tertiary care hospital in Karachi, Sindh, Pakistan, over a period of 10 years from January 2010 to December 2020. The study included patients of either gender who were over the age of 18 and had been diagnosed as having thyroid cancer on the basis of histopathology. Of these patients, the ones with insufficient data on immunohistochemical markers were excluded from our study. For the purposes of our study, the histological report of the tumor and the chemical markers found were assessed. The diagnosis of different tumors was established based on the biopsy of each tumor [7]. Immunohistochemical antibodies were applied on a semi-automated platform. Dilution is not required for all the clones to be ready to use. Table 1 describes clones against each immunohistochemical marker.

**Table 1:** Clones applied for each immunohistochemical marker. CEA: carcinoembryonic antigen, PAX-8: paired box gene-8, CD: cluster of differentiation, TTF-1: thyroid transcription factor-1. All the clones are ready to use, thus do not require dilution.

Immunohistochemical markers	Clones
Calcitonin	Polyclonal
Chromogranin	DAK-A3
Synaptophysin	DAK-SYNAP
CEA	Clone II-7
Cytokeratin AE-1	Clone AE1/AE3
Vimentin	Clone V9
PAX-8	MRQ-50
CD 68	PG-M1
CD 20	L-26
Ki 67	MiB-1
Thyroglobulin	Polyclonal
CD 56	Clone 123-C3
Cytokeratin 7	OV-TL 12/30
TTF-1	8 G7G3/1

Statistical methods

All statistical analyses were performed using the Statistical Package for Social Science (SPSS; Release 19.0, standard version, copyright^©^ SPSS; 1989-02, IBM Corp., Armonk, NY) and MS-Excel 2016. A descriptive analysis was performed with the mean ± standard deviation used to represent quantitative variables and percentages were used for qualitative variables. Proportions were used for categorical variables, while continuous variables were represented by mean ± standard deviation.

The diagnostic test measurements such as sensitivity, specificity, and positive and negative predictive value (PPV and NPV) were calculated for each variant and IHC marker.

## Results

Baseline demographics

Data on 124 thyroid cancer patients were collected based on the inclusion criteria; 68 were female and 56 were male. The mean age of the patients was 48.5 ± 15.6 years. Table 2 shows the distribution of various thyroid neoplasms in each gender.

**Table 2:** The histopathologic type of tumors and the distribution according to patient gender.

Histopathology of tumor	Male	Female	Total
Medullary	6	13	19 (15%)
Anaplastic	5	11	16 (13%)
Primary thyroid lymphoma	4	2	6 (5%)
Pure papillary	40	35	75 (61%)
Pure follicular	1	7	8 (6%)
Total	56	68	124

Thyroid carcinomas and immunohistochemical markers

Various immunohistochemical markers were used in our study, which is summarized in the tables below. Calcitonin was used in the majority of thyroid medullary cancer cases. Chromogranin and synaptophysin were also used in a few cases, as shown in Table 3. Cytokeratin AE-1, vimentin, paired-box gene-8 (PAX-8), and a cluster of differentiation 68 (CD68) have been applied in the cases of anaplastic thyroid carcinomas as shown in Table 4. In the cases of primary thyroid lymphoma, Cluster of Differentiation 20 (CD20) and marker of the proliferative index (Ki-67) have been used as depicted in Table 5. The most frequent immunohistochemical marker used was thyroglobulin, which has been applied to 80 of the total tumors, comprising papillary and follicular variants. In the papillary cancers, the cluster of differentiation 56 (CD56) has been used along with thyroglobulin, the negativity of the former is sensitive for this variant of lesions. Thyroglobulin has been applied as a tumor marker in follicular tumors. These are described in Table 6.

**Table 3:** Immunohistochemical markers in cases of medullary cancer of thyroid (n=19). CEA: carcinoembryonic antigen.

Immunohistochemical markers	IHC markers applied in cases of medullary cancer
Calcitonin	14
Chromogranin	9
Synaptophysin	7
CEA	4

**Table 4:** Immunohistochemical markers in cases of anaplastic cancer of thyroid (n=16). PAX-8: paired box gene-8, CD68: cluster of differentiation 68.

Immunohistochemical markers	IHC markers applied in cases of anaplastic cancer
Cytokeratin AE-1	11
Vimentin	9
PAX-8	2
CD68	3

**Table 5:** Immunohistochemical markers in cases of primary thyroid lymphoma (n=6). CD20: cluster of differentiation 20, Ki-67: marker of proliferative index.

Immunohistochemical markers	IHC markers applied in cases of primary thyroid lymphoma
CD 20	6
Ki-67	5

**Table 6:** Immunohistochemical markers in cases of papillary (n=75) and follicular (n=8) cancers of the thyroid. CD56: cluster of differentiation 56, CD20: cluster of differentiation 20.

Immunohistochemical markers	Papillary	Follicular
Thyroglobulin	73	7
CD56	22	0
Cytokeratin 7	12	2
Cytokeratin AE-1	4	3
CD20	7	0

Table 7 shows the sensitivity and specificity of some of the tumor markers commonly used for diagnostic purposes in thyroid tumors. Calcitonin was found to be a highly specific and sensitive marker for medullary carcinoma. Vimentin was a tumor marker highly specific and sensitive for anaplastic tumors of the thyroid while cytokeratin AE 1 was found to be sensitive. For thyroid lymphoma, Ki-67 was found to be specific and sensitive while CD20 was found to be highly sensitive. The papillary carcinoma uses thyroglobulin as a tumor marker which is highly sensitive but not specific, CD56 was used in one-third of the cases of papillary cancers where its negativity was highly sensitive, as it was not applied in other variants so specificity could not be assessed. For follicular tumors, thyroglobulin is found to be sensitive.

**Table 7:** Sensitivity, specificity, positive predictive values, and negative predictive values. PAX-8: paired box gene-8, CD20: cluster of differentiation 20, Ki-67: marker of proliferative index, CD56: cluster of differentiation 56.

Variant/IHC markers	Sensitivity	Specificity	Positive predictive value	Negative predictive value
Medullary carcinoma				
Calcitonin	1.00	0.75	0.93	1.00
Chromogranin	0.90	1.00	0.90	1.00
Synaptophysin	0.88	0.50	0.875	0.50
Anaplastic				
Vimentin	0.95	0.91	0.90	1.00
PAX-8	0.67	0.44	0.50	0.33
Primary thyroid lymphoma				
CD20	1.00	0.64	0.55	1.00
Ki-67	0.83	0.95	0.90	0.93
Papillary carcinoma				
Thyroglobulin	0.97	0.56	0.91	0.82
CD56	1.00	N/A	1.00	N/A
Follicular carcinoma				
Thyroglobulin	0.88	0.12	0.90	0.98

## Discussion

Currently, for the purpose of the diagnosis of a thyroid lesion, histological examination of Hematoxylin and Eosin (H&E) stained sections is used. However, the interpretation of various lesions can be quite challenging [8]. An example is the case of malignant follicular carcinoma versus benign follicular lesion, where the difference between the two is based on capsular and/or vascular invasion. This can prove to be challenging due to incomplete capsular penetration and difficulties in processing and sectioning the samples. Thyroid nodules are a fairly common clinical finding, and fortunately, most of these nodules turn out to be benign lesions. However, it is important to reach an accurate diagnosis in order to provide appropriate treatment. Modalities like thyroid ultrasound or fine-needle aspiration biopsy (FNAB) can be used to identify various lesions, but the reliability of these methods in differentiating between benign and malignant lesions is limited, resulting in most of these patients undergoing surgical resection even though only 10% of these patients have malignant tumors [9]. Accurate diagnosis is also important post-operatively as inaccuracy in interpretation can not only cause psychological and social stress but also unnecessarily increase the cost of healthcare for the patient [10].

Keeping these problems in mind, studies focusing on the diagnosis of different thyroid lesions by immunohistochemical markers have been carried out [7,11]. Identification of these markers for accurate diagnosis would be a huge benefit not only to the patient but also to the healthcare system, ensuring that patients receive the necessary and appropriate treatment, where they are managed surgically when needed and medically when appropriate [12].

Thyroglobulin is an iodoglycoprotein crucially involved in the synthesis of thyroid hormones, triiodothyronine (T3), and thyroxine (T4). In our study, we have found thyroglobulin to be a specific and sensitive tumor marker in cases of differentiated carcinoma of thyroid origin, namely papillary and follicular cancers. Several studies in the past have been done showing the significance of thyroglobulin in these tumors [13]. It is difficult to tell the difference between the follicular variant of papillary thyroid cancer (FVPTC) and other thyroid lesions with a follicular pattern. The lesions for which FVPTC cannot be ruled out are classified as well-differentiated tumors of unclear malignant potential (WDT-UMP) [14]. Mesothelial cell immunostain (HBME-1), galectin-3, and cytokeratin-19 (CK-19) are the most common IHC markers used in differential diagnosis. However, none of these markers can be used to make a 100% accurate differential diagnosis [15]. The cluster of differentiation 56 (CD56) negativity was discovered to be the only marker with a sensitivity and specificity of more than 90% (sensitivity 91.1% and specificity 91.7%). The most sensitive and specific dual panels were CD56 negativity and galectin-3 positivity (sensitivity 96 percent, specificity 85 percent), and the most sensitive and specific mixed panels were CD56 negativity, HBME-1 positivity, and galectin-3 positivity (sensitivity 96%, specificity 85%) (97% and 70%, respectively) [15]. In our study, CD56 negativity and thyroglobulin positivity were used in the diagnosis of PTC and follicular variant lesions. HBME-1 and galectin-3 have not been used in our center.

Thyroid transcription factor (TTF-1) is a homeodomain-containing transcription factor that controls the expression of certain genes involved in the morphogenesis of not only the thyroid but the lungs and central nervous system (CNS) as well. Therefore, its role as a tumor marker has not only been discussed in previous studies in thyroid tumors but malignancies of other systems as well [16].

Thyroglobulin and TTF-1 expression are lost in cases of anaplastic cancer [17]. However, in our study, TTF-1 was positive in 3 out of 16 cases and negative in the rest of the cases. Paired box gene 8 (PAX-8) retains expression in de-differentiated thyroid cancers and helps differentiate them from other head and neck neoplasms that are not separated due to poorly differentiated histomorphology [17]. Calcitonin is a serum marker highly specific for medullary carcinoma, involved not only in diagnostic but also in monitoring therapeutic results and recurrence. Studies have shown several medullary carcinomas that are calcitonin negative and highly aggressive. In our study, however, we found it to be a specific and sensitive serum marker for medullary carcinomas.

Cytokeratin AE-1 is a clone of anti-cytokeratin monoclonal antibodies used in detecting certain high and low molecular weight keratins, namely cytokeratin 10, 14, 15, 16, and 19 [18]. Its levels are used as a marker in several carcinomas, and in our study, we found it to be relatively sensitive and specific for anaplastic carcinomas. Vimentin is a multi-factorial protein involved in the migration of cancer cells that have undergone epithelial-mesenchymal transition [19]. It has also been found to play a role in various epithelial cancers [20]. In cases of thyroid cancers in our study, it was found to be highly specific and sensitive for anaplastic cancer. The cluster of differentiation 20 (CD20) is a cell surface marker present on T and B cells [21]. We found it to be a sensitive biomarker for thyroid lymphoma. A marker of the proliferative index (Ki-67) was used in cases of primary thyroid lymphoma to assess prognosis.

Strengths and limitations

The study, to the best of our knowledge, was the first of its kind performed on a Pakistani population with a large number of patients for papillary thyroid carcinoma. The study looked at a wide number of tumor markers that have been linked to thyroid tumor etiology in the past.

Our study was limited by the number of patients that had been treated over a 10-year period. In some of the cases, the associated risk factors for the patients with concurrent medical conditions could not be determined. The overall number of patients was 124, and the data being single-centric means that future multi-centric studies with a larger patient population would be needed to confirm the validity of our results.

## Conclusions

In our study, it can be concluded that calcitonin is a sensitive as well as a specific tumor marker in the cases of medullary carcinoma. Calcitonin-negative medullary carcinoma of the thyroid can be encountered; synaptophysin and chromogranin are used to supplement the diagnosis. PAX-8 can be used to differentiate de-differentiated lesions of thyroid origin from other head and neck neoplasms. Vimentin was found to have a sensitivity of 95% and a specificity of 91% in anaplastic cancers. The CD20 and a marker of the proliferative index (Ki-67) are used for primary thyroid lymphoma. Thyroglobulin is a reliable tumor marker raised in both papillary and follicular tumors but is not specific.

Tumor markers with good sensitivity and specificity can be used to help differentiate difficult histomorphologies, specifically in the cases of poorly differentiated carcinomas. However, to confirm and validate our results, studies on a larger sample of patients would be required.
